# Contradictory findings on one-year mortality following ICU delirium

**DOI:** 10.1186/s13054-015-0747-6

**Published:** 2015-01-30

**Authors:** Alison E Turnbull, Karin J Neufeld, Dale M Needham

**Affiliations:** Division of Pulmonary and Critical Care Medicine, Johns Hopkins University School of Medicine, 1830 E. Monument St – 5th Floor, Baltimore, MD 21205 USA; Outcomes After Critical Illness and Surgery (OACIS) Group, Johns Hopkins University, 1830 E. Monument St – 5th Floor, Baltimore, MD 21205 USA; Department of Psychiatry and Behavioral Sciences, Johns Hopkins University School of Medicine, 600 North Wolfe Street, Phipps 160, Baltimore, MD 21287 USA; Department of Physical Medicine and Rehabilitation, Johns Hopkins University School of Medicine, 600 North Wolfe Street, Meyer 1-130, Baltimore, MD 21287 USA

In contrast to prior studies (Table 1), Wolters and colleagues [1] reported no significant association between delirium and 1-year survival among Dutch ICU survivors. The authors attributed this finding to a novel adjustment of their Cox survival model for the sum of patients’ Sequential Organ Failure Assessment (SOFA) scores (obtained three times daily). Hence, we wish to enquire about the results of their multivariable model if re-run without SOFA adjustment: is the adjusted hazard ratio for delirium statistically significant? This contradictory result also could be attributable to other issues, rather than this unique adjustment that combined SOFA score and length of stay, as described below.

**Table 1:** **Selected studies evaluating mortality after ICU delirium**

	**Significant association between delirium and mortality**						**No association**
	**Ely:**	**Kiely:**	**Pisani:**	**Van Rompaey:**	**Shehabi:**	**Abelha:**	**Wolters:**
	***JAMA***	***JAGS***	***AJRCCM***	***J Clin Nutr***	***Crit Care Med***	***Crit Care***	***Crit Care***
**Year**	2004	2009	2009	2009	2010	2013	2014
**Location**	USA	USA	USA	Netherlands	5 countries	Portugal	Netherlands
**Study population**	224 ventilated MICU and CCU patients	412 ICU survivors with delirium at discharge	304 MICU patients	105 non-intubated ICU patients	354 ventilated ICU patients	562 SICU patients with non-emergency surgery	1,101 ICU patients
**Patient characteristics at admission**							
**Age (mean (sd))**	Del = 56 (17)	84 (7)	75 (9)	62	62 (15)	Median (IQR)	60 (17)
	No del = 54 (17)					Del = 65 (54-74)	
						No del = 64 (53-73)	
**Severity of illness (mean (sd))**	APACHE II	NR	APACHE II minus CNS component	APACHE II	APACHE II	Median (IQR)	APACHE IV
	Del = 26(8)			Del = 26(8)	19 (7)	APACHE II	61 (29)
	No del = 23(10)		22 (6)	No del = 23(10)		Del = 9 (7-12)	
						No del = 8 (5-10)	
**ICU mortality**	13%	NR	16%	7%	NR	1%	13%
**ICU LOS (median (IQR))**	NR	NR	5	Del = 13.4	Del = 16 (7 - 23)	Del = <1 (<1–3)	Del = 8 (5 – 15)
				No del = 2.5	No del = 4 (3 - 6)	No del = <1 (<1- <1)	No del = 3 (2-5)
**Delirium measure**	Binary; time-dependent	Persistence; time-varying	Days; time-varying	Binary	0,1,2, ≥3 days	Binary	Binary
**Mortality assessment**							
**Time (months)**	6	12	12	3 and 6	1	6	12
**Mortality in ICU survivors**	NR	39%	41%	11% (3 mo)	NR	13%	18%
				12% (6 mo)			
**Model**	Cox survival	Discrete time survival	Cox survival	Logistic	Cox survival	Logistic	Cox survival
**Results (95% CI)**	HR = 3.2	HR = 2.9	HR = 1.1	OR 3 and 6 mo:	HR 1,2, ≥3 versus	OR = 2.6	HR = 1.26
	(1.4-7.7)	(1.9-4.4)	(1.02-1.18)	4.3 (1.3-14.7)	0 days:	(1.4-4.8)	(0.93-1.71)
				3.8 (1.1-13.1)	1.7 (1.3-2.3)		
					2.7 (1.6-4.6)		
					3.7 (1.9-7.2)		

APACHE, Acute Physiology and Chronic Health Evaluation; CCU, coronary ICU; CI, confidence interval; CNS, central nervous system; Del, delirium; HR, hazard ratio; HRQoL, health-related quality of life; IQR, interquartile range; LOS, length of stay; MICU, medical intensive care unit; mo, months; NR, not reported; OR, odds ratio; sd, standard deviation; SICU, surgical ICU.

The study’s 1-year mortality was 18%, with 59% of survivors discharged home. In contrast, 1-year mortality in a prior US study was 41% [2], raising the question of whether Dutch ICU survivors are healthier than those in some prior studies? Perhaps delirium is more strongly associated with mortality in frail ICU survivors. The Dutch study also reported that delirium was associated with increased cognitive impairment without impaired quality of life. Consequently, we wonder if care for cognitively impaired individuals is better in the Netherlands, hence conferring less risk of mortality to ICU survivors with cognitive impairment.

Finally, what were the results of testing the proportional hazards assumption in the Cox model? If the association between delirium and mortality is time-dependent [3], with the hazard greatest shortly after discharge, as per a prior Dutch study [4], a single hazard ratio over 1 year could attenuate this measure and violate the proportional hazards assumption.

## Authors’ response

Annemiek E Wolters and Arjen JC Slooter

We appreciate the opportunity to respond to the thoughtful comments of Dr Turnbull and colleagues.

In our cohort of ICU survivors, we did not find an association between delirium during ICU stay and 1-year mortality. We re-ran the analysis without adjusting for cumulative SOFA scores, and still did not find an association. The difference with other studies must therefore be attributable to other factors, including selection of the population [1]. It should be noted that most studies included ICU patients but were not restricted to survivors of ICU stay, as ours was.

Other explanations for the difference in findings between our study and others could be that the association between delirium and mortality may only be present in frailer former ICU patients, and that aftercare differed between our study and previous investigations. In addition, earlier studies with higher mortality rates included, on average, older patients [2,5]. Furthermore, our study included ICU survivors from 2009 to 2011 [1]. The studies with higher mortality rates were conducted at least 5 years earlier, and these participants could not have benefited from similar improvements of care over time [2,5].

The proportional hazard assumption was not violated. The log-minus-log plot showed nearly parallel lines. Also, the interaction term between a function of survival time and delirium was not significant (*P* = 0.33), meaning that the effect of delirium on mortality did not vary significantly with time.

## Abbreviation

SOFA: Sequential organ failure assessment
